# Classification of Sporting Activities Using Smartphone Accelerometers

**DOI:** 10.3390/s130405317

**Published:** 2013-04-19

**Authors:** Edmond Mitchell, David Monaghan, Noel E. O'Connor

**Affiliations:** Centre for Sensor Web Technologies, Dublin City University, Dublin D9, Ireland; E-Mails: david.monaghan@dcu.ie (D.M.); noel.oconnor@dcu.ie (N.E.O.)

**Keywords:** smartphone, classification, sport

## Abstract

In this paper we present a framework that allows for the automatic identification of sporting activities using commonly available smartphones. We extract discriminative informational features from smartphone accelerometers using the Discrete Wavelet Transform (DWT). Despite the poor quality of their accelerometers, smartphones were used as capture devices due to their prevalence in today's society. Successful classification on this basis potentially makes the technology accessible to both elite and non-elite athletes. Extracted features are used to train different categories of classifiers. No one classifier family has a reportable direct advantage in activity classification problems to date; thus we examine classifiers from each of the most widely used classifier families. We investigate three classification approaches; a commonly used SVM-based approach, an optimized classification model and a fusion of classifiers. We also investigate the effect of changing several of the DWT input parameters, including mother wavelets, window lengths and DWT decomposition levels. During the course of this work we created a challenging sports activity analysis dataset, comprised of soccer and field-hockey activities. The average maximum F-measure accuracy of 87% was achieved using a fusion of classifiers, which was 6% better than a single classifier model and 23% better than a standard SVM approach.

## 1. Introduction

Smartphone usage has grown dramatically since their introduction over a decade ago. Over 50% of adults in the United States and over 40% of adults in Europe own a smartphone [1]. By 2016 it is expected that there will be one billion smartphone owners worldwide [2]. A smartphone is a mobile phone with a purposely built mobile operating system with advanced computing ability and interconnectivity compared with a standard mobile phone. Smartphones have more advanced Application Programming Interfaces (APIs) for running third party applications. They also contain technology that standard phones lack, such as portable media players, digital cameras, GPS navigation systems and modern web browsers. One key feature provided by smartphones relevant to this work is access to embedded sensors, such as gyroscopes, magnetometers and accelerometers.

When a series of wirelessly networked sensors are positioned on a human body, this is referred to as Wireless Body Area Networks (WBANs). Any sensor that can gather some form of physiological data can be integrated into a WBAN. Examples include piezoresistive sensors to measure breathing rate [3] and electrocardiograms used to measure heart rate. Physiological sensors integrated into a WBAN can be used for computer assisted rehabilitation [3], diagnosing illnesses and providing care for incurable conditions. WBANs promote non-invasive wireless monitoring, which allows patients to have increased levels of freedom. Subjects can be monitored constantly for symptoms at home, work or while on hospital grounds. WBANs also allow physiological changes in athletes to be monitored while they are in their preferred environment. For instance, foot pressure insole technology allows track athletes to record foot pressure in their natural environment *i.e.*, on the track, whereas previously pressure plate technology required the athlete to be in an unfamiliar scientific setting [4].

Physiological data collected from WBANs can be used to ascertain the state and activity of a person independent of external infrastructure. This is called human activity recognition and it is important in various applications such as monitoring the health and security of the elderly who live alone for example, with a goal of improving their quality of life, freedom and safety [5]. One application that is beginning to receive attention is the benefit that activity recognition technology can give to athletes [6]. Here activity recognition can help athletes gather performance metrics quickly and easily, help physiotherapists identify possible injury concerns and give coaches detailed information on their players' fitness and ability.

Much of the research completed in WBAN activity recognition has dealt with detecting everyday tasks such as eating, ascending and descending staircases, sitting, brushing teeth as well as motion activities such as walking, jogging and running [7–9]. Additionally much of this research employs multiple strategically placed sensors that can detect individual limb movement. However additional sensors significantly increase the cost of the system. By utilizing data available from smartphones, the comparable cost is significantly reduced, only requiring users to download an appropriate software application and a cheap garment for securing their smartphone.

Accelerometers have been used for human activity recognition in a large amount of existing work [10–12]. Research has shown that accelerometers can be used to identify human activity for high energy actions such as walking, jogging, jumping, *etc.* [13]. In sports, accelerometers have been used to monitor elite athletes in competition or training environments. In swimming applications, accelerometers have allowed the comparison of stroke characteristics for a variety of training strokes and therefore have helped to improve swimming technique [14]. When used in competitive rowing and coupled with other monitoring techniques such as impeller velocity, they allow for the recovery of intra- and inter-stroke phases as a means to assess performance and this has been used by competition rowers to improve performance at national and international competitions [10].

Most approaches in human activity recognition have relied on multiple expensive sensors. With the increase in smartphone ownership there has been more research conducted utilizing the sensors embedded within smartphones. Human activity recognition using smartphones have been employed to support patient monitoring [15], to identify the user's current mobility [16] and for monitoring daily activities [17]. However in this work we will show how smartphones can be used to recognize human activity in sport. To the best of our knowledge this is the first such study conducted.

In any activity recognition problem, feature extraction is a vital operation to determine those features with relatively small intra-class yet large inter-class variations that can be used as the basis for effective classification. It is preferable to have a low number of features due to the associated reduction of the computational load of the classification process. One method to extract discriminative features from a signal is to use the wavelet transform. The wavelet transform splits a signal into different frequency components, and then analyses each component with a resolution matched to its scale. Wavelets have advantages over traditional Fourier methods in analyzing physical situations where the signal contains discontinuities and sharp spikes.

**Figure 1.**
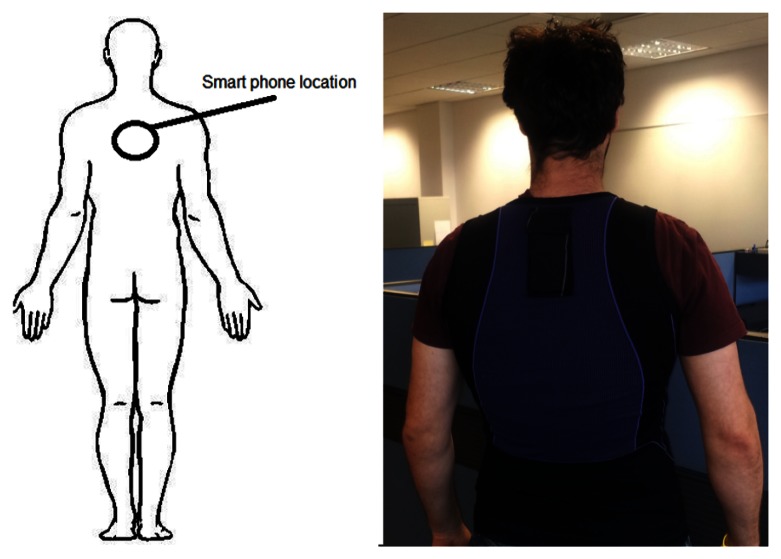
Location of Smartphone.

In this paper, we take advantage of the embedded accelerometer within a smartphone and position it on the upper crevice of a user's back as seen in Figure 1 to classify sporting events. There is a large amount of literature for activity recognition but it is limited for classifying sporting activities. Most of this literature uses custom albeit commercially available sensors requiring athletes at any level to purchase these sensors such as the miCoach and the Nike+. However virtually everyone already has a smartphone in their pocket. Smartphone ownership in the United States increased by 5% to 110 million from February to May 2012 alone and this trend appears to continue [18]. Performing classification using the smartphone potentially makes the technology available to everyone at all levels without additional hardware but a cheap vest.

Whether or not player monitoring technology is allowed in competition varies from sport to sport. Both low-cost solutions, e.g., miCoach or Nike+, and high-end offerings, e.g., GPSports, are used widely at all levels in training sessions and competition (when allowed). However, in both cases the level of automatic data analysis provided for understanding player activity is quite limited. Our technology can be considered to be a low-cost solution that provides finer grained information about players' activity based on an automatic classification framework.

Athletes can take advantage of this technology to judge their overall match and training participation, physiotherapists could be notified of potential injuries and coaches could factor this information into their team selection. In sports where the wearing of sensors is forbidden during competitive matches, this technology can still be used in training environments to access an athlete's performance. We set our sample rate to a low value that current smartphones can easily accommodate (16–22 fps) when logging raw accelerometer signals.

Since the Discrete Wavelet Transform (DWT) correlates the input signal with a mother wavelet function, the choice of mother wavelet function is a important activity that has a significant impact on the performance of any application using wavelets. Similarly the amount of times the DWT decomposes a signal, referred to as the DWT level, has a direct impact on performance. The length of time chosen to separate activities is called the window length and has a direct effect on classification accuracy. Therefore, in this work, we also examine the effect of contrasting types of mother wavelet functions, chosen DWT level and window length on classification performance and compare performance in each case.

## 2. Targeted Activities and Experimental Methodology

We captured accelerometer data from two different field sports, five-a-side soccer and field hockey. Hockey players regularly change their back position when performing field hockey activities. For this reason field hockey was chosen as an alternative to the more difficult five-a-side soccer. Five-a-side soccer was chosen as it was envisaged that it would present significant difficulty in attempting to recognize activities. This was due to the smartphone being placed upon the upper area of a user's back as shown in Figure 1. Players wearing the vest reported that wearing it did not affect their performance due to the placement of the smartphone and the light weight nature of the vest.

In all forms of soccer the primary appendage used is the feet, therefore deciphering actions executed by the feet from the upper back presents a difficult challenge. Consequently, achieving accurate results with five-a-side soccer is an ideal challenge for our classification approach. Seven different sporting activities common to both five-a-side soccer and field hockey are targeted for classification. In this context, an activity is defined as a quantifiable action preformed by the user that is deemed significant. With this definition we identify the following activities:

A1 Subject is stationary (0) m/s
A2 Subject is walking (1 ± 1) m/s
A3 Subject is jogging (3.5 ± 1.5) m/s
A4 Subject is sprinting (5+) m/s
A5 Subject is hitting the ball
A6 Subject is attempting a standing tackle
A7 Subject is dribbling the ball

We believe these activities to be comprehensive yet generic as they cover both inertial (A1–A4) and game (A5–A7) activities. Table 1 displays the specification of the smartphones employed to collect our dataset. From experimental observation we have found that the constant recording of the sensors on a smartphone of this nature for a period of 1 hour uses approximately 20% of the battery. When the dataset was being constructed, these smartphones were at the more expensive range of the smartphone market. Cheaper smartphones with less advanced hardware would not be able to capture accelerometer data at a high rate so we chose the standard rate for sampling so that in principle any smartphone could be used.

Five-a-side soccer data was recorded during five matches with each lasting one hour. From these five matches, the accelerometer data from 15 players was recorded. For field hockey, six matches were recorded with a total of 17 different players. Each match was video recorded with a Sony DCR-SR50, which allowed player activities to be accurately annotated by synchronising the video data with the accelerometer data. When logging an activity, nine seconds of data was collected, with the activity being placed in the centre of this window. This allowed us to experiment with different window sizes for feature extraction. Nine seconds was chosen as it was large enough for these sporting activities to be completed and small enough that it did not drastically increase computational time.

Datasets were created that contained 30 examples of each activity from both five-a-side soccer and field hockey data. These 30 examples were chosen randomly from activities logged from the matches recorded. These datasets contained activities from various players and allowed the comparison based on varying classification model parameters. In activity classification problems, one important aspect is the changes in performance when different people perform the same activity. This inter-subject variability can have a distinct effect on classification accuracy. Each individual performs an activity differently due to their weight, height, sex and strength. In this study we captured data from a variety of players in order to get a realistic classification result. Subjects whose data was captured while playing soccer were amateur enthusiasts, whereas subjects whose data was captured while playing field hockey were elite athletes. By capturing data in a naturalistic environment, we reduce the possibility that a player's activities have been altered by the experiment.

## 3. Feature Extraction

Once data is captured, features must be extracted that will allow the identification of activities. Features with higher correlation between similar patterns (intra-class variation) and poorer correlation between dissimilar patterns (inter-class variation) are desirable. Analysis with a large number of variables can require a large amount of computer memory and computation power. More importantly, a large input into a classification algorithm can cause overfitting in the training sample, which produces models that respond poorly to new samples. Choosing the most discriminative features is key; otherwise, the model will not be able to distinguish between samples and the classification accuracy will be unsatisfactory.

### 3.1. The Wavelet Transform

The wavelet transform has been used with much success in extracting discriminative features from data that aid in classification [19–21]. The wavelet transform is a commonly used function [22] in signal processing applications such as decomposing, compression, feature extraction, encoding, and signal reconstruction. The Fourier transform is the cornerstone of discrete signal processing due to its ability to deal with linear time-invariant operators or uniformly regular signals but for signals that have transient properties, the Fourier transform becomes a cumbersome tool that requires a large number of coefficients to represent a localized event. Wavelet bases, like Fourier bases, reveal the signal regularity through the amplitude of coefficients, and their structure leads to a fast computational algorithm. However, wavelets require few coefficients to represent local transient structures because they are well localized. The technical computing software MATLAB [23] has toolboxes that allow for the extraction of DWT coefficients from a data signal.

With the Discrete Wavelet Transform (DWT), any signal can be decomposed into a group of discrete wavelet coefficients. Almost all DWTs use filter banks for the analysis and reconstruction of a signal that may contain either finite impulse response or infinite impulse response filters. The filter banks contain high and low frequency filters to derive the frequency content of the signal in the sub-bands. Therefore the DWT decomposes a discrete signal into two sets of coefficients; approximation and detail. It then subsamples the resulting signals by two.

**Figure 2.**
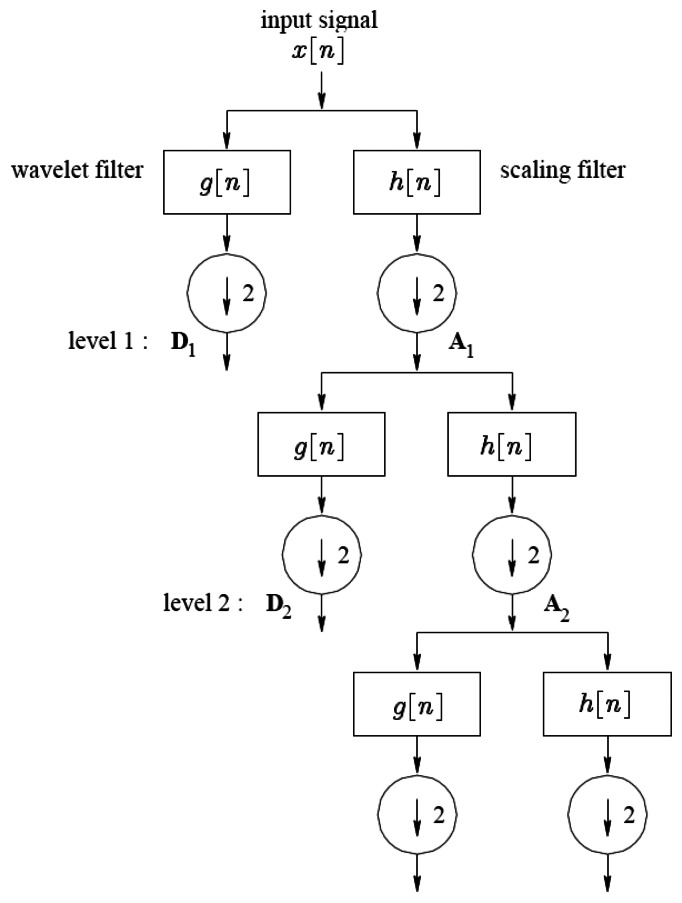
DWT decomposition of signal *x*[*n*].

Using the same method, the resulting approximation coefficients are then split into new approximation and detail coefficients. This procedure is iteratively executed to create a group of approximation coefficient vectors *A_i_* and detail coefficient vectors *D*_1_, *D*_2_,…, *D_i_* at the *i*th level, as outlined in Figure 2. There are $\frac{N}{2^{i}}$ elements in the approximation vector *A_i_* and $\frac{N}{2^{i}}$ elements in the detail vector *D_j_* (where *j* = 1,…, *i*) when the original signal has *N* elements.

The choice of mother wavelet is crucial as it generates all the wavelet functions that determine the properties of the resulting wavelet transform, which in turn relates to the transform's performance in any application. Currently there is no standardized way to select the mother wavelet and the choice depends on the application. The most important and commonly considered parameters when choosing a wavelet are its amounts of vanishing moments, its regularity, compactness and symmetry.

In signal processing the most commonly used wavelets are Haar, Daubechies, Coiflet, Symlet, bi-orthogonal and reverse bi-orthogonal. Coiflets and Symlets evolved from the Daubechies wavelet. Daubechies, Coiflet and Symlet are orthogonal and compactly supported wavelets. Daubechies wavelet is asymmetric, compactly supported and has minimum-phase associated scaling filters. Coiflet is near symmetric, compactly supported and has the highest number of vanishing moments. Symlet has the least asymmetry, compactly supported and has linear-phase associated scaling filters. These wavelets suffer poor regularity.

### 3.2. Energy of the DWT at Each Level

In this work, features of player accelerometer signals are extracted using the DWT and fed into a classification algorithm in order to correctly identify a player's current sporting activity. DWT decomposition levels ranging from one through seven are investigated. Further levels would increase computational time significantly. The total energy *E_T_* at level *i* of the DWT decomposition is given by [8]:

(1) $$E_{T}=A_{i}A_{i}^{T}+\sum_{j=1}^{i}D_{j}D_{j}^{T}$$

where *A_i_* is the approximation coefficient at level *i* and *D_i_* is the detailed coefficient at level *i*. One feature that can give discriminating results is the energy ratio in each type of coefficient [8]. *EDR_A_* represents the energy ratio of the approximation coefficients while *EDR_Dj_* represents the energy ratio of the detail coefficients.

(2) $$EDR_{A}=\frac{A_{i}A_{i}^{T}}{E_{T}}$$

(3) $$\begin{array}{cc} EDR_{D_{j}}=\frac{D_{i}D_{j}^{T}}{E_{T}} & j=1,\ldots ,i \end{array}$$

With the EDRs calculated, a foundation has been created for detailed information features to be extracted. In [8] Ayrulu-Erdem and Barshan found that the normalized variances of the DWT decomposition coefficients and the EDRs provided the most informative features for a different albeit similar problem. They contrasted their performance to informational features such as normalized means, minimums and maximums of the EDRs and obtained superior performance. As such we adopt the same approach here. The variances of the coefficients are calculated over each DWT coefficient vector at the *i*th level

(4) $$A_{i},D_{1},D_{2},D3,\ldots ,D_{i}$$

**Figure 3.**
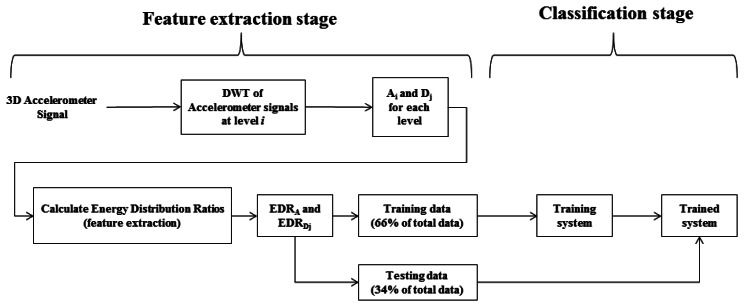
System overview of the DWT decomposition and classification process.

Therefore, at the *i*th level there are *i* + 1 variance values calculated for each axis segment, totalling 3(*i* + 1) features for an accelerometer signal. The amount of EDR features is equal to the amount of DWT coefficients. Adding these features to the variances gives a total of 6(1 + *i*) features at level *i*. Figure 3 gives a system overview of both the feature extraction stage and classification stage.

## 4. Classifiers Investigated

In the previous section we explained how we extract features that can be used to train a classifier. A classifier refers to a mathematical function that maps input data to a category. In this section, we discuss five popular families of classifiers that were employed for sports activity classification using the discrete wavelet transform decomposition of accelerometer signals. John C. Platt's Sequential Minimal Optimization (SMO) optimization algorithm was used for the training of the support vector machine (SVM) classifier. The IBk classifier is a simple instance-based learner that uses the k-nearest neighbour (k-NN) algorithm for training. The Naive Bayes classifier applies Bayes' theorem with strong (naive) independence assumptions to train its classification models. A logistic model tree (LMT) [24] is a decision tree with logistic regression functions at the leaves for supervised learning tasks. A multilayer perceptron (MLP) is a feedforward artificial neural network that utilizes back-propagation for training a network.

### 4.1. Support Vector Machine (SVM)

Support vector machines have secure theoretical foundations, strong regularization properties and excellent empirical successes. They have been applied to tasks such as image classification [25], speech processing [26], protein classification [27] and human activity classification [28]. Support vector machines can be defined as systems that use hypothesis space of linear functions in a high dimensional feature space, which are trained with a learning algorithm from optimization theory that implements a learning bias derived from statistical learning theory [29]. SVM performs well on data sets that have a large amount of attributes, even data sets that contain very few cases on which to train the model. In fact there is no upper limit on the number of attributes a data set can contain and hardware poses the only constraints. The SMO algorithm is used to efficiently solve the optimization problems that occur during the training of SVMs. SVMs are often described as a “black box” classifier as the user doesn't need to choose many parameters.

### 4.2. K-Nearest Neighbour (K-NN)

K-NN algorithms are used for classifying data based on closest training examples in the feature space. K-NN is a class of instance-based learning techniques where the function is only approximated locally and calculations are suspended until classification. The K-NN algorithm finds a group of *k* objects in the training set that are nearest to the input object, and judges the allocation of a label on the predominance of a class in this neighbourhood. For this method there are three basic components: a set of labelled attributes, a distance measure to compute distance between objects, and the value of *k*, the number of nearest neighbours. To classify unknown data, the distance of this data to the known data is computed, its k-nearest neighbours are determined, and the class labels of these neighbours are then used to identify the class label of the unknown object. K-NN algorithms can handle missing values, are robust to outlying data points, and have a good history as predictors. They tend to only handle numeric variables, are sensitive to monotonic transformations of features, are not immune to insignificant inputs, and provide models that are difficult to interpret. The K-NN algorithm sets equal weighting to all inputs; therefore, it is sensitive to noise and redundant features. It has been used in many applications in the field of data mining, statistical pattern recognition, image processing and many others. Some successful applications include recognition of handwriting [30], text classification [31] satellite imagery analysis [32] and ECG pattern analysis [33].

### 4.3. Bayesian Network

One very important probability-band classifier is the naive Bayes method, which is also known as idiot's Bayes, simple Bayes, or independence Bayes. It assumes that the presence or absence of a particular feature of a class is unrelated to the presence or absence of any other feature, given the class variable. This method is significant for many reasons. It does not need any complicated iterative parameter estimation schemes; therefore, it is simple to construct. This means it may be applicable to large datasets. One advantage of the naive Bayes classifier is that to calculate the parameters (means and variances of the training data) required for classification, it only requires a small amount of training data. Only the variances of the variables for each class need to be determined because independent variables are assumed, and not the whole covariance matrix. This classifier has been used in a large range of applications such as medical diagnosis [34], data mining [35] and musical style recognition [36].

### 4.4. Classification Tree

Classification trees create a model that predicts the value of a target variable based on several input features. In these tree structures, leaves represent class labels and branches represent conduits that allow features to lead to class labels. A logistic model tree, which is a classification tree with logistic regression functions at the leaves, was employed for this work. This method has been shown to give better results [24] than standard decision trees and simpler logistic methods. A stage-wise fitting process is used that selects relevant attributes in the data. This incrementally refines the leaves constructed at higher levels in the tree. The logistical model tree has been used in applications such as ECG arrhythmia studies [37], textual entailment classification [38] and real-time human movement classification using accelerometers [11].

### 4.5. Artificial Neural Network

Biological neural networks have inspired mathematical models called artificial neural networks (ANNs). A multilayer perceptron (MLP) [39] is a feedforward ANN that consists of multiple layers of nodes that each have the same destination, with each layer completely connected to the adjacent layers. Apart from the input and output nodes each node is a neuron, that is to say, a processing element with a nonlinear activation function. MLP uses backpropagation for training the network, which allows the network to converge on a satisfactory feature weighting and flow. MLP is an adaptation of the standard linear perceptron and can analyse data that is not linearly separable. The MLP has been used in a wide array of classification problems such as skin segmentation [40], classification of multispectral satellite images [41] and recognizing human motion with multiple acceleration sensors [9]

### 4.6. Classification Methodology

We created four datasets with 210 sporting activities. Multiple classification models were generated from these datasets. Two thirds of the dataset was used as training data and the remaining data was used as testing data. We randomized the data and calculated each model's accuracy a total of 10 times. We then took each of these ten calculated accuracies and took the average as the model's overall accuracy. We believe that this gives a fair and balanced view of the model's accuracy. Choosing a value over ten did not change the result significantly but resulted in a significant increase in computational time. The highest accuracy models were tested with a 10-fold cross-validation for additional validation.

F-measure gives a measure of a test's accuracy. It uses both precision *p* and recall *r* of a test to calculate the score. Precision is calculated as the number of correct results divided by the number of total results, while recall is the number of correct results divided by the number of results that should have been returned positive. These metrics are often described in terms of the metrics true positive (*T_p_*), false positive (*F_p_*) and false negative (*F_n_*). The F-measure score is a harmonic mean of precision and recall, where an F-measure score reaches its best value at 1 and worst score at 0. In this work all results are presented using the F-measure algorithm.

(5) $$F_{1}=2.\frac{\text{Precision}\times \text{Recall}}{\text{Precision}+\text{Recall}}$$

(6) $$\begin{array}{cc} \text{Precision} & =\frac{T_{p}}{\left(T_{p}+F_{p}\right)}\\ \text{Recall} & =\frac{T_{p}}{\left(T_{p}+F_{n}\right)} \end{array}$$

## 5. Results and Discussion

### 5.1. Benchmarking

In Reference [42], Kwapisz *et al.*, extracted forty-three time domain features from a smartphone accelerometer and employs them, along with a ANN for activity recognition. These activities are walking, jogging, walking upstairs, walking downstairs, sitting and standing. They achieved an overall recognition accuracy of over 90%. Due to this high result, we employed Kwapisz *et al.*,'s methods on our dataset. It achieved an average accuracy rate of 73% for soccer and 79% for field hockey. It took 2 ms to compute the time domain features for a ten second data window.

As mentioned earlier, the Fast Fourier Transform (FFT) is a popular method for extracting informational features from a data signal. In Reference [21] Preece *et al.*, used FFT techniques along with a k-NN classifier for activity recognition. These activities are walking, jogging, walking upstairs, walking downstairs, running, hopping on left leg, hopping on right leg and jumping. We also employed these extraction techniques on our dataset. It achieved an average accuracy rate of 78.1% for soccer and 78.5% for field hockey. It took 25 ms for the FFT features to be extracted using MATLAB from a ten second data window. All computation duration tests in this work were completed on an Intel Core 2 Quad CPU Q9650 processor with 4 gigabytes of RAM.

### 5.2. Experiment 1

A black box experiment uses a system that is viewed solely based on the input and output. In this scenario parameters are selected based on their popularity or their ease of use. SVMs are one of the most popular classifiers used in human activity problems as it is relatively simple to understand and quick. Therefore for this baseline experiment we used an SVM and have selected the parameters in Table 2. Daubechies 4 wavelet “db4” is a popular mother wavelet choice in signal analysis problems due to its regularity and fast computational time. A level two DWT was chosen to keep computational time short while still extracting discriminative features. A window length of five seconds was chosen as every activity had concluded by then. It took 10 ms for the DWT features to be extracted from the 5 second window. It took 4 ms for this approach to classify the extracted DWT features with the SVM.

For field hockey this experiment achieved a 65.9% F-Measure score while for soccer it achieved a 62.7% F-Measure score. Field hockey and soccer models both suffered from high mean absolute error, 21.13% and 21.41% respectively. Tables 3 and 4 give the confusion matrix for this experiment. Both models identify inertial activities (A1–A4) adequately, but perform poorly when trying to identify game activities (A5–A7). This approach is the fastest to create and train, however this is outweighed by its relatively poor performance compared with other approaches.

### 5.3. Experiment 2

In experiment 2 we investigate the full range of classifiers and vary the input parameters to understand to what extent they influence the classification procedure. We inspect how adjusting the classifier, DWT decomposition level, window length and mother wavelet affects our framework.

**Figure 4.**
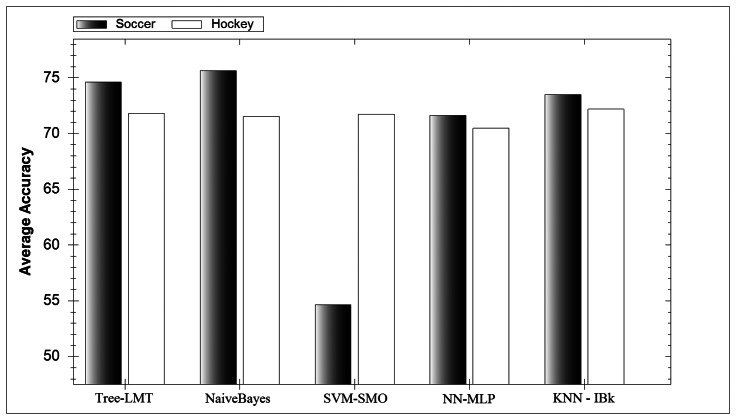
Average classifier family accuracy for experiment 2.

In Figure 4 the average accuracy for each classifier investigated can be seen. Interestingly all classifiers perform similarly expect for the SVM-SMO classifier during soccer. Further investigation showed that this classifier could not reliably distinguish between lower extremity game activities. SVM classifiers themselves have many parameters and therefore require tweaking to get their full potential. During hockey activity classification the SVM-SMO performed well as the game activities were much more distinct. Interestingly the average soccer model outperforms its hockey counterpart, which was not expected. A reason for this could be that the range of parameters investigated favoured soccer classification. However the highest accuracy hockey models created performed better than the highest accuracy soccer models, which was expected.

**Figure 5.**
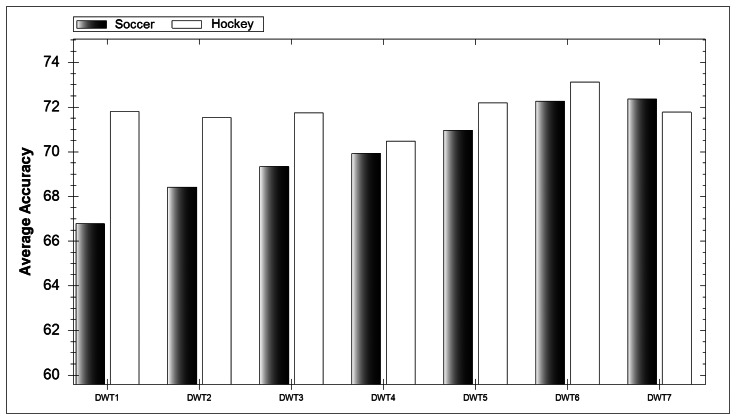
Effect of DWT Levels on classification accuracy.

In Figure 5 the overall average accuracy for each DWT level over all classifiers can be observed. It is interesting to note that there is an increase in average accuracy with every level increase during soccer while during hockey classification each level performs similarly well. As mentioned earlier, five-a-side soccer was envisaged to be much more difficult to classify due to the position of the smartphone. Therefore we can conclude that retrieving more features with additional decomposition levels allows classifiers to rectify difficult-to-interpret data.

**Figure 6.**
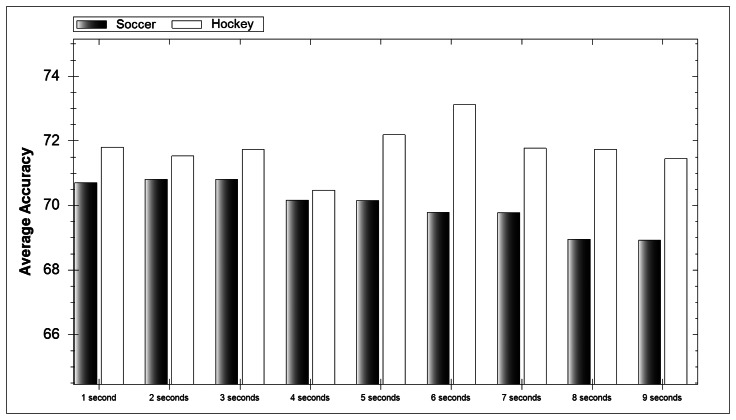
Effect of window length on average accuracy.

In Figure 6 the accuracy average for each window length can be seen over all classifiers. For soccer the accuracy of the model decreases with an increase in window length. This makes sense as soccer activities have a shorter duration than their hockey counterparts. With shorter activities the longer the window the more activities can occur. If two or more activities occur in a window then classification difficulty is dramatically increased. When selecting a time window it is vital that it is long enough to contain the whole activity being performed and short enough that it does not include additional events.

**Figure 7.**
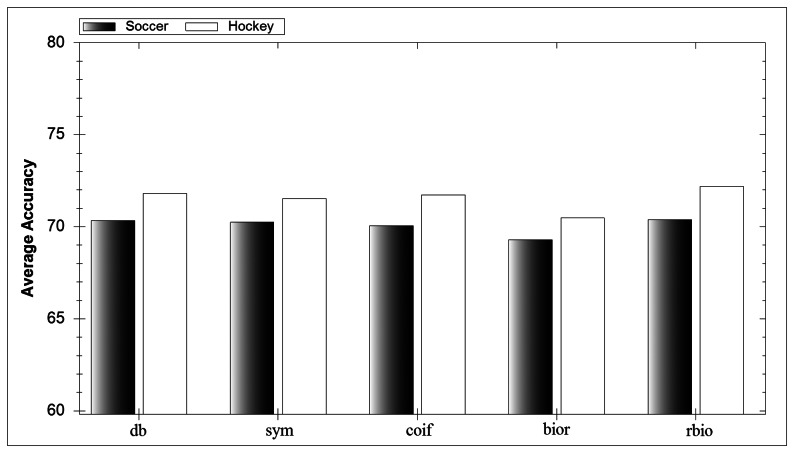
Effect of choice of wavelet.

In Figure 7 the average accuracy for each mother wavelet family can be seen. Each family performs well and no one family outperforms the rest. This result reinforces the belief in the literature that it is almost impossible to prejudge what mother wavelet will perform well in an application. Similarly, results from individual wavelets show no discernible difference between their performances. However the mother wavelet itself is important due to its integral part in the DWT process.

As with Experiment 1, classifiers are very competent at identifying inertial movement activities (A1–A4), but game activities (A5–A7) pose more of a challenge. The inertial activities are very distinct as the energy during these activities is unique. They range from zero energy output when the player is stationary to maximum energy when the player is sprinting. The confusion encountered between the game activities is due to the similar motions being performed. In soccer these motions involve lower leg movement while in hockey these game activities involve the upper arm movement. Table 5 provides the parameters for the highest classification accuracy attained for each respective sport. Tables 6 and 7 display their confusion matrix data. It took 5 ms for this approach to classify the extracted DWT features.

### 5.4. Experiment 3

**Figure 8.**
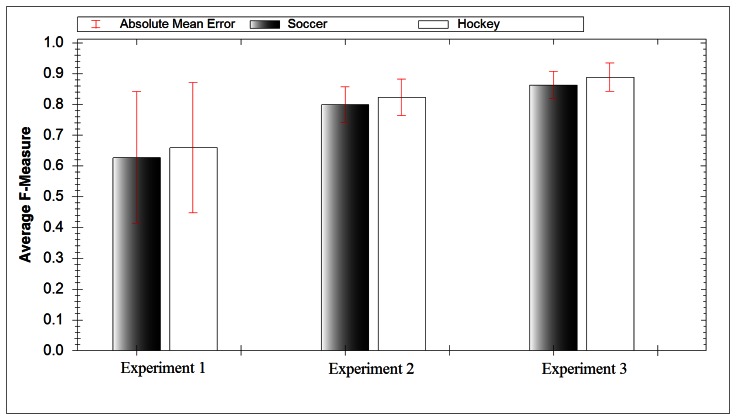
Average model accuracy for each experiment.

**Figure 9.**
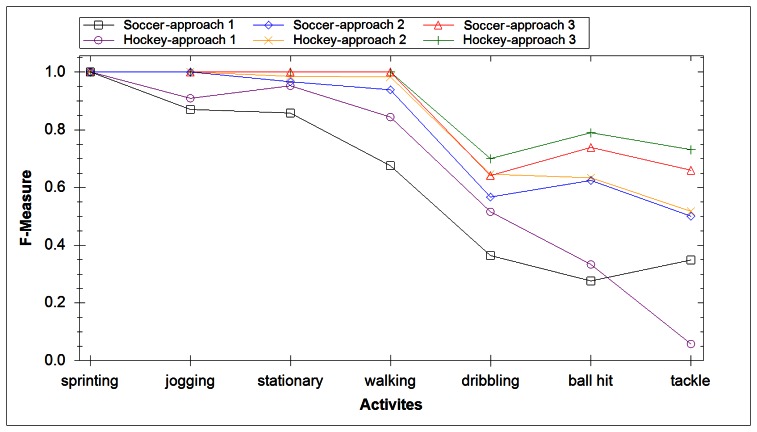
Single activity accuracy results for each approach.

In our final experiment we investigated creating a separate classification model for each activity. This allows us to create a fusion of classifiers whereby the classifier result with highest confidence dictates the result. The average F-measure score for soccer data yielded a result of 86.3%, while for hockey data it yielded 88.8%. Both results had low mean absolute error, 4.42% for football and 4.55% for hockey. Figure 8 compares the accuracy of all three experiments and includes the absolute mean error. Figure 9 compares the ability of each experiment at identifying specific activities. It took 27 ms for this approach to classify the extracted DWT features. The increased computational time compared with experiment 2 is due to testing the extracted DWT features of a signal with each activity model.

## 6. Conclusion

In this paper we present a framework that allows for the automatic identification of sporting activities from a single smartphone worn on the upper body. We extract discriminative informational features from smartphone accelerometer signals using the Discrete Wavelet Transform (DWT) decomposition. These features were very informative as they were able to reduce accelerometer signals to a much less complex input. For example with the first model in Table 8 we were able to reduce the accelerometer segment from 75 samples into 42 descriptive features. Training and classifying activities would take much longer and be prone to over-fitting without reducing the signal to a small set of features. One disadvantage of using the DWT when extracting features from signals is that there is no widely accepted method of picking the most suitable mother wavelet for a particular application. We investigated five prominent mother wavelet classes, *i.e.*, Daubechies, Coiflets, Symlets, Biorthogonal and reverse Biorthogonal. Daubechies provided the mother wavelet for half of the best accuracy models. However, the overall results show that performance differences between mother wavelets are not very significant.

No one classifier family has to date been shown to have a direct advantage in activity classification problems so we examined classifiers from each. Investigating different window lengths, DWT level, classifier and mother wavelet allowed us to create classification models that achieved F-measures of 86.3% and 88.8% for five-a-side soccer and field hockey respectively. This DWT extraction process is much more accurate compared with state-of-the-art time domain and FFT feature extraction methods as seen in Section 5.1.

In this work we also investigate the effect of changing several of the DWT input parameters, including mother wavelets, window lengths and DWT decomposition levels. During the course of this work we created a unique sports activity analysis dataset, comprised of five-a-side soccer and field hockey activities. All our experimental results presented in this paper are based on this dataset. The average maximum F-measure accuracy of 87% was achieved using a fusion of classifiers, which was 6% better than a single classifier model and 23% better than a standard SVM approach. However this relatively modest 6% increase comes with significant increase in computation.

Most approaches in human activity recognition rely on multiple expensive sensors. With the increase in smartphone ownership, there has been more research conducted utilizing the sensors embedded within smartphones. Human activity recognition using smartphones have been employed to support patient monitoring [15], to identify the users current mobility [16] and for monitoring daily activities [17]. In this work we have shown that smartphones can be used to recognize human activity in sport.

Performing classification using data gathered by a smartphone potentially makes the technology available to everyone at all levels without additional hardware but a cheap vest. Currently all processing is performed offline after data gathering. If real time processing is required then our preferred solution would be a continuous connection to a server rather than performing the analysis on the smartphone itself. Our software supports real-time data streaming, but we have not implemented real-time analysis to date—this is targeted for future work.

The approach proposed here for human sporting activity classification could be applied to other human motion activity problems. Now that the framework has been set up, the key problem when creating classification models is acquiring sufficient training data. Additionally, this method is not confined to an offline setup, especially with smart phones that possess the ability to communicate over the web. The smartphone also has many other embedded sensors that could be used to capture physiological information. Future work will focus on investigating this and also comparing other feature extraction methods with the DWT. We also will investigate other feature dimensionality reduction techniques such as principal component analysis. Furthermore, there are other sensors that have grown in popularity such as the miCoach by Adidas. It would be interesting to investigate their performance compared with smartphones [43].

## Figures and Tables

**Table 1.** Smartphone specifications.

**Smartphones Used**		

	**Google Nexus One**	**HTC Desire**
Sampling Rate	16 Hz	25 Hz
Accelerometer	Tri-axial	Tri-axial
Resolution	8-bit	8-bit

**Table 2.** Parameter specifications for black-box approach.

**Classifier**	**Mother Wavelet**	**DWT Level**	**Window Size**
SVM-SMO	db4	2	5 seconds

**Table 3.** Confusion matrix for Soccer Smartphone data for Experiment 1.

**Activity**	**A1**	**A2**	**A3**	**A4**	**A5**	**A6**	**A7**
A1	30	0	0	0	0	0	0
A2	3	27	0	0	0	0	0
A3	0	0	30	0	0	0	0
A4	0	0	0	30	0	0	0
A5	0	15	3	0	7	4	1
A6	4	4	2	0	7	8	5
A7	3	4	4	0	7	4	8

**Table 4.** Confusion matrix for Hockey Smartphone data using Experiment 1.

**Activity**	**A1**	**A2**	**A3**	**A4**	**A5**	**A6**	**A7**
A1	30	0	0	0	0	0	0
A2	3	27	0	0	0	0	0
A3	0	0	30	0	0	0	0
A4	0	0	0	30	0	0	0
A5	0	6	5	0	11	1	7
A6	0	1	0	0	16	1	12
A7	0	0	1	0	9	3	17

**Table 5.** Highest classification accuracies attained for Experiment 2.

**Device**	**Sport**	**Classifier**	**DWT lvl**	**Mother W.**	**Length (Sec)**	**F-Measure**
Smartphone	Soccer	NaiveBayes	6	rbio1.1	3	0.799
Smartphone	Hockey	MLP	6	bior1.1	7	0.823

**Table 6.** Confusion matrix for Football Smartphone data for Experiment 2.

**Activity**	**A1**	**A2**	**A3**	**A4**	**A5**	**A6**	**A7**
A1	28	0	0	0	0	0	0
A2	0	30	0	0	0	0	0
A3	0	0	30	0	0	0	0
A4	0	0	0	30	0	0	0
A5	0	1	0	0	24	4	1
A6	0	2	0	0	9	12	7
A7	0	1	0	0	12	2	15

**Table 7.** Confusion matrix for Hockey Smartphone data for Experiment 2.

**Activity**	**A1**	**A2**	**A3**	**A4**	**A5**	**A6**	**A7**
A1	30	0	0	0	0	0	0
A2	1	29	0	0	0	0	0
A3	0	0	30	0	0	0	0
A4	0	0	0	30	0	0	0
A5	0	0	0	0	19	7	4
A6	0	0	0	0	7	15	8
A7	0	0	0	0	4	6	20

**Table 8.** Highest classification accuracies attained.

**Device**	**Sport**	**Classifier**	**DWT lvl**	**Mother W.**	**Length (Sec)**	**F-Measure**
Smartphone	Soccer	NaiveBayes	6	rbio1.1	3	0.799
Smartphone	Hockey	MLP	6	bior1.1	7	0.823
